# Discoidin domain receptor 1a (DDR1a) confers 5-fluorouracil cytotoxicity in LoVo cell via PI3K/AKT/Bcl-2 pathway

**DOI:** 10.1080/21655979.2022.2060782

**Published:** 2022-04-13

**Authors:** Bin Xiong, Fei-Xue Song, Hui-Ling Chen, Xiao-Juan Wang, Zheng-Xu Jin, Ti-Yun Han, Yi Li, De-Kui Zhang

**Affiliations:** a Department of Oncology, The Second Hospital of Lanzhou University, Lanzhou, Gansu, China; b The Second Clinical Medical College, Lanzhou University, Lanzhou, Gansu, China; c Laboratory of Digestive Disease, The Second Hospital of Lanzhou University, Lanzhou, Gansu, China; d School/Hospital of Stomatology Lanzhou University, Lanzhou, Gansu, China; e Department of Gastroenterology, The Second Hospital of Lanzhou University, Lanzhou, Gansu, China

**Keywords:** Discoidin domain receptor 1a, colorectal cancer, proliferation, cytotoxicity, 5-fluorouracil

## Abstract

5-Fluorouracil (5-FU) is a common chemotherapy drug for patients with advanced colorectal cancer; however, many patients develop resistance to 5-FU and suffer from treatment failure. Discoidin domain receptor 1 (DDR1) is upregulated in multiple cancers and positively associated with chemoresistance. We explored the effect of DDR1a on the cytotoxicity induced by 5-FU in LoVo cells and the underlying mechanism. Therefore, DDR1a overexpression (DDR1a^high^) and knockdown in LoVo cell lines (shDDR1a) were constructed to detect cell viability and cytotoxicity induced by 5-FU. The results showed that cell viability of DDR1a^high^ cells was higher in comparison with that of the control group. When 5-FU (5 µM) was administered, the percentage of apoptotic cells, cytochrome C release and caspase-3 activity was found to be higher in the shDDR1a group than that in the control group. Both of PI3K and MDM2 proteins level decreased in DDR1a^high^ and shDDR1a, but the BAX/Bcl-2 level in the shDDR1a group increased compared to that in the control. Therefore, DDR1a might be a potential therapeutic target for 5-FU chemoresistance in colorectal cancer.

## Graphical abstract

**Figure:**
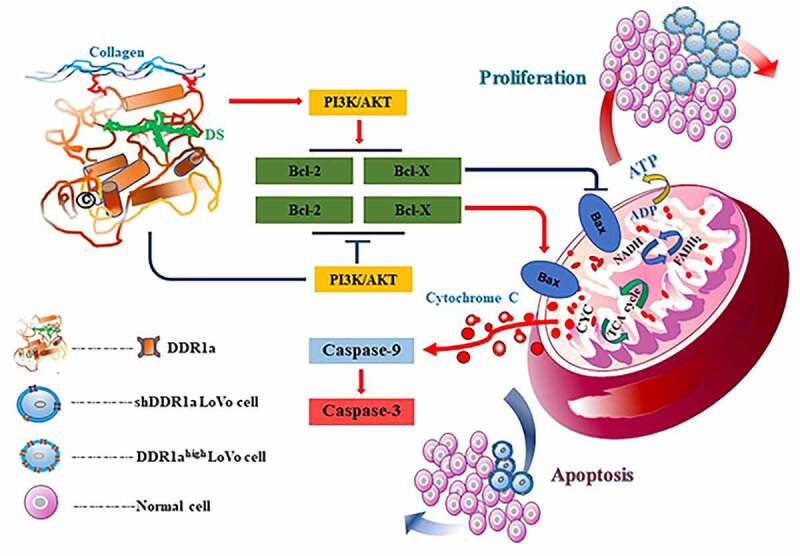

## Introduction

Colorectal cancer (CRC) was one of the major causes of cancer*‐*related morbidity and mortality globally [1]. 5-fluorouracil (5-FU) is an anti-metabolite chemotherapeutic drug with fluorine substituted for hydrogen at the uracil C-5 position [2]. Systemic 5-FU has been used for decades, and remains the drug used in first-line therapies such as CRC chemotherapy [3]. It is currently used in combination with irinotecan, oxaliplatin and folinic acid. Unfortunately, 5-FU resistance limits its clinical effectiveness and leads to relapse and poor patient prognosis. Therefore, it is of great significance to explore the mechanism by which CRC cells develop resistance to 5-FU, and identify novel treatment strategies to improve patient outcomes.

Discoidin domain receptor 1 (DDR1), a novel tyrosine kinase receptor, was identified by its catalytic kinase domains and activated by various collagens [4,5]. DDR1a is one of the five DDR1 isoforms that is generated by alternative splicing [6]. Recent studies have shown that DDR1 dysfunction is a consequence of mutations, and is upregulated in various cancers, including lung [7], gastric [8], prostate [9], glioma [10] and hepatocellular [11]. Moreover, DDR1 upregulation is associated with poor prognosis and resistance to genotoxic drugs [12]. Extensive studies have revealed that DDR1 regulates various biological and cellular processes of tumors, including proliferation, differentiation, migration, and invasion [5,6,12,13]. However, the exact molecular mechanisms of DDR1 in tumorigenesis and chemoresistance remain unclear.

In this study, we hypothesized that DDR1a may play an important role in 5-FU chemoresistance. To test and validate this hypothesis, lentivirus-mediated knockdown and overexpression of DDR1a was performed, then the effects of DDR1a and/or 5-FU on LoVo cell proliferation, apoptosis, and its regulatory mechanism were investigated. Our study provides novel insights into CRC chemoresistance.

## Materials and methods

### Cell culture and treatment

The human colorectal carcinoma cell line LoVo, provided by Prof. You-Cheng Zhang (Department of General Surgery, Second Hospital of Lanzhou University, Lanzhou, China) was cultured in ATCC-formulated Ham’s F12K Medium, supplemented with 10% fetal bovine serum (Gibco, Thermo Fisher Scientific Inc. Waltham, MA, USA), 100 U/mL penicillin, and 100 μg/mL streptomycin in a humidified atmosphere containing 5% CO_2_ at 37°C.

### DDR1a knockdown/overexpression in LoVo cells

shDDR1a and DDR1a^high^ cells were constructed to clarify the role of DDR1a in proliferation and sensitivity of 5-FU-induced cytotoxicity in LoVo cells. The LV3-DDR1a-homo-1043 and LV3-DDR1a-homo-1733 vector with sequences 5′-GGCTGGATGACTTTAGGAAGA-3′ and 5′-GGGACACTATCCTCATCAACA-3′, respectively, were designed to target the DDR1a knockdown in LoVo cells. Furthermore, LV3-DDR1a shRNA (LV3) with sequence 5′-TCTCCGAACGTGTCACGT-3′ was used as a negative control. The LV3-DDR1a-homo-1043 vector was selected to construct shDDR1a using quantitative polymerase chain reaction and western blot analysis. Furthermore, LoVo cells were transfected with lentivirus-mediated DDR1a vectors carrying an anti-puromycin gene with a green fluorescent protein. Subsequently, LoVo cells were infected with recombinant lentiviruses for 72 h using a medium containing 1.2  μg/mL puromycin to screen positive transfected cell lines in each group, with the involvement of DDR1a^high^-NC and shDDR1a-NC control vectors.

### CCK-8 assay

The cell viability and cytotoxicity induced by 5-FU were measured using the CCK-8 assay (Dojindo, Kumamoto, Japan) [14]. First, the transfected LoVo cells were seeded in 96-well plates at 5 × 10^3^ cells/well. Then, various concentrations of 5-FU were added to obtain an optimal concentration for evaluating the effect of DDR1a on the cytotoxicity induced by 5-FU. Following this, 10 μL of CCK-8 solution was added to each well at 24, 48, 72, and 96 h. Finally, the cells were incubated for 2 h according to the manufacturer’s instructions, and the absorbance was measured at 450 nm using an automatic spectrophotometer PowerWave X (Bio-Tek, Santa Clara, CA, USA). The IC_50_ was calculated using SPSS version 22.0 (SPSS Inc., Chicago, IL, USA).

### Bromodeoxyuridine (BrdU) assay

The proliferation of LoVo cells was measured using BrdU cell proliferation enzyme-linked immunosorbent kit (ab126556, Abcam, Cambridge, UK) [15]. LoVo cells were counted and seeded in 96-well plates at 1 × 10^4^ cells/well. BrdU was added to each well and incubated at 37°C in a humidified atmosphere containing 5% CO_2_ for 24 h, the cells were then fixed in a fixing solution (200 μL/well) for 30 min and incubated with anti-BrdU antibody (100 μL/well) for 2 h at room temperature. Next, peroxidase goat anti-mouse IgG (100 μL/well) was added to the cells and incubated for 30 min at room temperature. Finally, the absorbance was measured at 530 nm using an automatic spectrophotometer PowerWave X (Bio-Tek, Santa Clara, CA, USA).

### Hoechst 33,342 staining

The apoptosis induced by 5-FU was evaluated using the Hoechst 33,342 staining kit, and the nuclear morphology was observed after the transfected cells were treated with 5 and 10 μM 5-FU at 48 and 72 h [16]. Briefly, each cell group was fixed with 3.7% paraformaldehyde at room temperature for 15 min and washed three times with phosphate-buffered saline (PBS). Then, cells were stained with 10 mg/L of the Hoechst 33,342 stain in the dark, and at room temperature for 15 min. Morphological changes of apoptotic nuclei were observed under fluorescence microscope BX53F (Olympus Corporation, Tokyo, Japan), and cells with condensed chromatin or shrunken, irregular, or fragmented nuclei were considered apoptotic. The percentage of apoptotic cells was calculated by counting the number of apoptotic cells in five random fields per slide.

### Terminal deoxynucleotidyl transferase (TdT) dUTP nick-end labeling (TUNEL) assay

TUNEL assay (Roche, San Francisco, CA, USA) was used to confirm the effect of DDR1a on apoptosis induced by 5-FU [17]. First, transfected LoVo cells were seeded at 5 × 10^3^ cells/well and incubated in a medium containing 5-FU (5 and 10 µM) for 48 h. Next, the medium was removed and incubated in equilibration buffer, TdT enzyme, anti-digoxigenin peroxidase conjugate, and TMB substrate solution for 20 min at 37°C in the dark. The cells exhibiting red fluorescence were defined as TUNEL-positive apoptotic cells under the fluorescence microscope BX53F (Olympus Corporation). The percentage of TUNEL-positive cells was calculated from five random fields in three slides.

### Cytochrome C enzyme-linked immunosorbent assay (ELISA) assay

The release of cytochrome C from mitochondria was determined using the Cytochrome C ELISA Kit (Invitrogen, Carlsbad, CA, USA) according to the manufacturer’s instructions [18]. LoVo cells were seeded at 5 × 10^3^ cells/well and incubated in a medium containing 5 µM 5-FU for 48 or 72 h. Next, the medium was removed and incubated with a cell lysis buffer on ice for 10 min. Then, the lysates were centrifuged at 10,000 × *g* for 5 min; a reaction buffer was added to each well and incubated for 3 h at room temperature. Next, biotin-conjugated, diluted streptavidin–horseradish peroxidase and 3,3′,5,5′-tetramethylbenzidine (TMB) substrate solution was added to all the wells. Finally, the absorbance was read at 600 nm using an automatic spectrophotometer PowerWave X (Bio-Tek, Santa Clara, CA, USA).

### Caspase-3 activity assay

The caspase-3 activity was determined with the caspase-3 Assay kit (Jiancheng Bioengineering Institute, Nanjing, China) [19]. Briefly, the cells were collected and washed twice with cold PBS. The cells were resuspended and lysed on ice for 30 min. The supernatant was incubated with Ac‐DEVD‐pNA and a reaction buffer at 37°C for 4 h. Next, the absorbance was measured at 405 nm using an automatic spectrophotometer PowerWave X (Bio-Tek, Santa Clara, CA, USA). Finally, we determined the protein concentration of the extracts using the Bradford assay.

### Western blot analysis

Proteins of cells were obtained according to this method [20]. The untreated or treated LoVo cells were washed three times with ice-cold PBS, and the protein was extracted using a RIPA lysis buffer containing 1 mM phenylmethylsulfonyl fluoride (PMSF). The protein concentration was detected using a bicinchoninic acid (BCA) protein assay kit (Beyotime Biotechnology, China), and 25 μg protein was separated using 10% sodium dodecyl sulfate–polyacrylamide gel electrophoresis gel electrophoresis and transferred onto polyvinylidene fluoride membranes. After blocking with 5% skim milk in Tris-buffered saline containing 0.1% Tween-20 (Sigma-Aldrich, St Louis, MI, USA) for 1 h at room temperature, the membranes were incubated with primary antibodies including anti-DDR1, anti-PI3K, anti-p-AKT, anti-AKT, anti-MDM2, anti-P53, anti-BAX, anti-Bcl-2, and anti-β-actin (1:1,000) at 4°C overnight. Next, the membranes were washed with Tris-buffered saline containing 0.05% Tween-20 (Sigma-Aldrich) thrice and incubated with peroxidase-conjugated secondary antibodies (1:3,000) for 2 h at room temperature. Finally, the membranes were developed using ECL chemiluminescence reagents (Amersham Pharmacia Biotech, Tokyo, Japan). The protein band intensities were determined using Quantity One software (Bio-Rad Laboratories, Hercules, CA, USA).

### Statistical analyses

All experiments were performed in triplicate with at least three independent experiments. Data are presented as the mean ± standard deviation. The statistical analyses were performed using the SPSS version 22.0 (SPSS Inc). The results were evaluated by one-way analysis of variance followed by Bonferroni’s multiple-comparison analysis. Statistical significance was set at *P* < 0.05.

## Results

The aim of this study was to investigate the role of DDR1a in 5-FU chemoresistance. Our results showed that DDR1a overexpression (DDR1a^high^) led to a higher proliferation rate and resistance to 5-FU. During the knockdown of DDR1a (shDDR1a) it exhibited typical apoptotic morphological features and stronger apoptotic fluorescence intensity with increasing concentration of 5-FU. Furthermore, after 5 μM of 5-FU treatment, the caspase-3 activity and cytochrome C release was upregulated in shDDR1a cells, accompanied by a lower Bax/Bcl-2 ratio. To summarize, the results suggested that DDR1a might play an important role in 5-FU chemoresistance.

## Overexpression of DDR1a promotes LoVo cell proliferation

**Figure 1.**
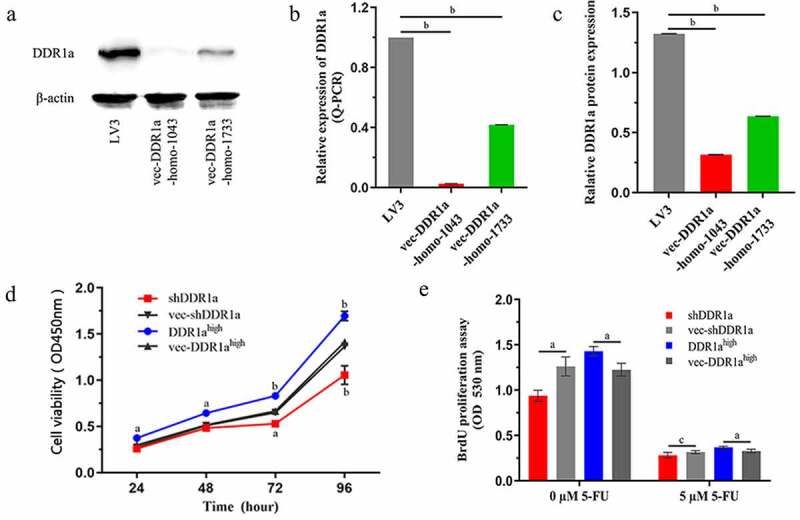
The effects of DDR1a knockdown and overexpression on cell proliferation. Two vectors targeted shDDR1a: LV3-DDR1a-homo-1733 , LV3-DDR1a-homo-1043 , and DDR1a overexpression (DDR1a^high^) were performed. LoVo cells were transfected with shDDR1a or DDR1a overexpression vector, LV3-DDR1a shRNA (LV3) or vec- DDR1a^high^ were negative control. (a-c) Transfection efficiency of Lentivirus-mediated knockdown of DDR1a were investigated by Q-PCR and western blot. ^b^*p*<0.01 compared with LV3. () Cell proliferation was determined using CCK-8 (d) and BrdU assay (e). ^a^*p* < 0.05, ^b^*p*<0.01, ^c^*p*>0.05 compared with vec-shDDR1a or vec-DDR1a^high^.

The LoVo cells were transfected with shDDR1a or DDR1a overexpression vectors, and transfection efficiency was assessed via Q-PCR or western blot (Figure 1a–c). The cell viability showed that LoVo cell proliferation had increased significantly by inducing DDR1a overexpression but decreased by DDR1a knockdown after 48 h (*P* < 0.05;Figure 1d). The results were also confirmed by BrdU assay (Figure 1e).

## Knockdown of DDR1a enhanced cytotoxicity induced by 5-FU in LoVo cells

**Figure 2.**
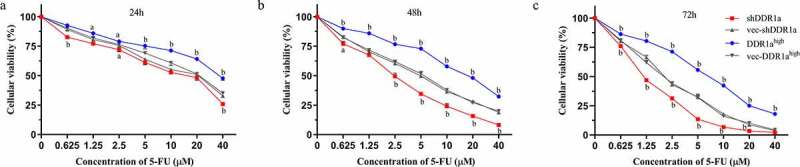
Knockdown DDR1a increases 5-FU sensitivity by inhibiting LoVo cell growth. Transfected cells were treated with 0–40 μM 5-FU for 24 h (a), 48 h (b), and 72 h (c), and the cytotoxicity was determined using CCK-8 assay. ^a^*p* < 0.05, ^b^*p* < 0.01 compared with vec-shDDR1a or vec-DDR1a^high^.

Using 5-FU inhibited cell growth in a concentration- and time-dependent manner (Figure 2). It was observed that the cell viability in both shDDR1a and DDR1a^high^ cells decreased with the increase in concentrations of 5-FU. However, Table 1 indicates that the IC_50_ of DDR1a^high^ cells (48 and 72 h) increased significantly compared with vec-DDR1a^high^(*P* < 0.01). The mean IC_50_ of 5-FU in DDR1a^high^ cells was six times higher than that in the shDDR1a cells (16.15 vs 2.59 µM). Thus, DDR1a knockdown enhanced the cytotoxicity of 5-FU.

**Table 1.** The IC_50_ of 5-FU in LoVo cells

5-FU 24 h 48 h 72 h	IC_50_	shDDR1a	vec-shDDR1a	DDR1a^high^	vec-DDR1a^high^
		10.55^c^ 2.59^a^ 1.31^a^	15.03 4.85 2.27	43.69^a^ 16.15^b^ 6.48^b^	17.91 5.13 2.19

^a^*p* < 0.05, ^b^*p*<0.01, ^c^*p*>0.05, compare with vec-shDDR1a or vec-DDR1a^high^.

## Knockdown of DDR1a aggravated apoptosis induced by 5-FU in LoVo cells

**Figure 3.**
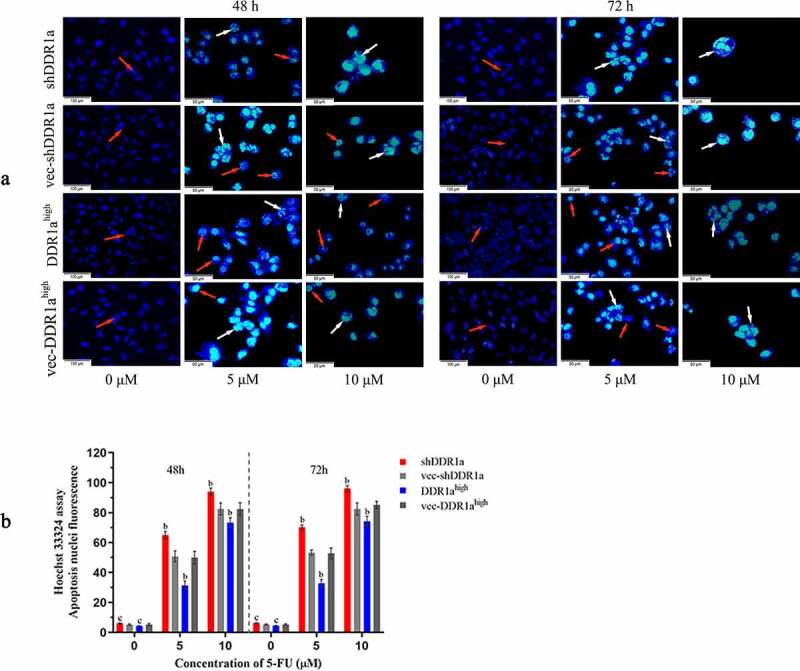
Effects of DDR1a knockdown and overexpression on apoptosis induced by 5-FU in LoVo cell. The apoptosis induced by 5-FU was determined using Hoechst 33,342 staining (2 μg/mL). Transfected cells were treated with 5 or 10 µM 5-FU for 48 and 72 h. The red arrows indicate normal cells and the white arrows indicate apoptotic cells. The cell fluorescence was detected by a fluorescence microscope (a, scale bar: 50 µm; ×20 magnification), and then image analysis was performed(b). ^b^*p*<0.01, ^c^*p*>0.05, compare with vec-shDDR1a or vec-DDR1a^high^.

**Figure 4.**
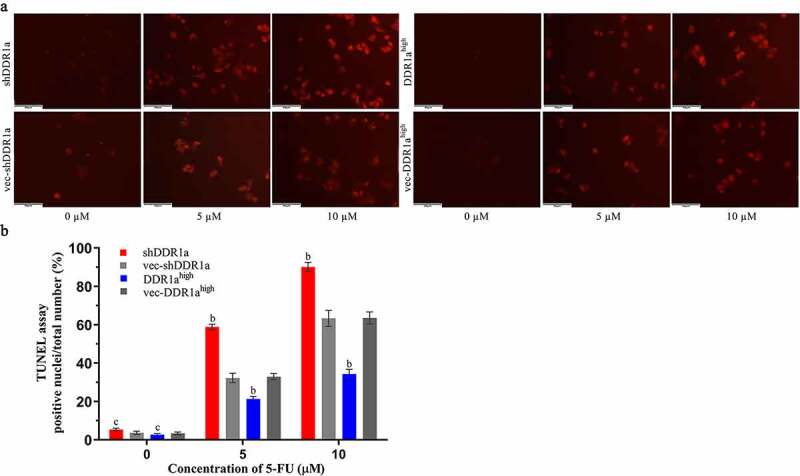
Effects of DDR1a knockdown and overexpression on apoptosis induced by 5-FU in LoVo cell. Transfected cells were treated with 5 and 10 µM 5-FU for 48 h, then the TUNEL assay was performed. The fluorescence intensity was detected by a fluorescence microscope (a scale bar: 50 µm; ×20 magnification), and was quantified by ImageJ software (b). ^b^*p*<0.01, ^c^*p*>0.05, compare with vec-shDDR1a or vec-DDR1a^high^.

The Hoechst 33,324 staining showed that transfected cells treated with 5 and 10 μM 5-FU for 72 h presented apoptotic characteristics of chromatin shrinkage, nuclear fragmentation, and massive vacuolation. Moreover, the apoptosis rate significantly increased in DDR1a knockdown cells compared with that in vector controls (Figure 3a, b). TUNEL assay also confirmed the results: the bar plot of the TUNEL positive cells shows that the number of apoptotic cells in the shDDR1a cells was about onefold higher than that of the control (58.82 ± 0.85 vs 32.28 ± 1.40 at 5 µM 5-FU, *P* < 0.01; 90.11 ± 1.38 vs 63.33 ± 2.40 at 10 µM 5-FU, *P* < 0.01) (Figure 4a, b). These results indicated that the knockdown of DDR1a promoted apoptosis induced by 5-FU.

## Knockdown of DDR1a facilitates cytochrome C release and caspase-3 activation

**Figure 5.**
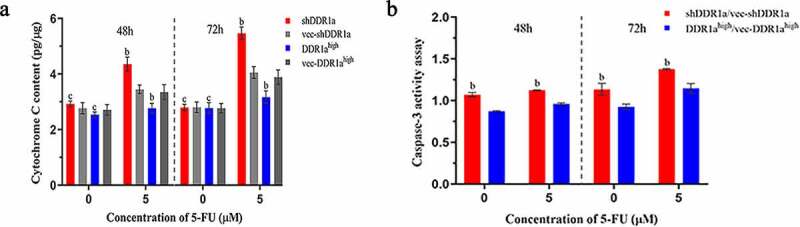
Knockdown DDR1a promotes 5-FU induced cytochrome C release and caspase-3 activation. (a) Cytochrome C release was determined by cytochrome C ELISA assay. (b) The caspase-3 activity assay in transfected cells. ^b^*p*<0.01, ^c^*p*>0.05, compare with vec-shDDR1a or vec-DDR1a^high^.

Because the release of cytochrome C from mitochondria triggers caspase activation, we then examined cytochrome C and activation of the initiator caspase 3. There were no statistical differences in cytochrome C among the DDR1a^high^, shDDR1a cells, and their negative controls (Figure 5a). However, cytochrome C content in the shDDR1a group was significantly higher than that in the control (4.36 vs 3.48, *P* < 0.01) after the treatment with 5-FU for 48 h. Meanwhile, DDR1a knockdown upregulated the caspase-3 activity; after treatment with 5-FU for 48 h, the caspase-3 activity increased compared with that in the control (1.13 vs 0.96 at 5 µM 5-FU, *P* < 0.05) (Figure 5b). Thus, DDR1a knockdown may be involved in cytochrome C release and caspase-3 activation mediated by 5-FU.

## DDR1a regulated PI3K/AKT/Bcl2 signaling pathway

**Figure 6.**
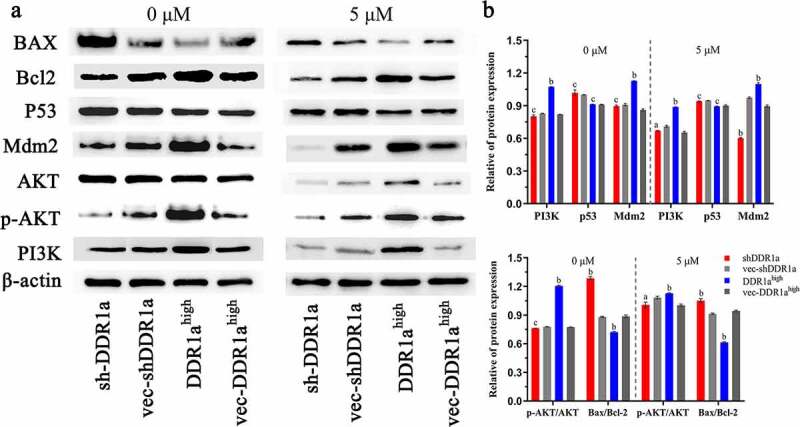
**DDR1a regulated PI3K/AKT/Bcl2 signaling pathway** (a) The transfected cells were treated with or without 5 μM 5-FU, then the Western blot was performed. (b) The bar chart represents the quantitative measurement of the relative proteins normalized with β-actin in three independent assays. ^a^*p* < 0.05, ^b^*p*<0.01, ^c^*p*>0.05, compared with vec-shDDR1a or vec-DDR1a^high^. PI3K, phosphatidylinositol 3-kinase; p-AKT, phosphorylation protein kinase B; AKT, protein kinase B; MDM2, Murine double minute 2; Bcl-2, B-cell lymphoma/leukemia-2; BAX, Bcl-2 associated X.

DDR1a overexpression promoted the expression of PI3K, MDM2 proteins, and upregulated the p-AKT/AKT ratio. There were no statistical differences in p53 expression among DDR1a^high^, shDDR1a, and their negative controls. However, DDR1a knockdown elevated the BAX/Bcl-2 ratio (1.49 vs 1.38, *P* < 0.05). After treatment with 5-FU (5 μM) for 48 h, the expression of PI3K and MDM2 proteins decreased in DDR1a^high^ and shDDR1a, but the BAX/Bcl-2 level in the shDDR1a group increased compared to that in the control (1.12 vs 0.95, *P* < 0.01) (Figure 6a, b). These findings illustrated that the PI3K/AKT/Bcl2 signaling pathway is regulated by DDR1a in LoVo cells.

## Discussion

Recent studies have shown that DDR1 function is associated with multiple cancers, and high DDR1 expression is correlated with poor prognosis in several tumors [8,21,22]. 5-FU is the most commonly used chemotherapeutic drug in patients with CRC. However, numerous CRC cases develop 5-FU resistance, which is a major challenge to the effective treatment of cancer [23]. The emergence of drug resistance depends on the genetic instability, heterogeneity, and high mutational rate of tumor cells among others. Among the 11 established CRC cell lines, LoVo cells are characterized by homozygous deletion of the *p53* gene, with deficiency in *p53* mRNA transcription, and show microsatellite instability [24,25]. Therefore, we chose LoVo cells for this study. The CCK-8 assay demonstrated that overexpression of DDR1a promoted cell proliferation while knockdown of DDR1a inhibited cell proliferation in LoVo cells. Thus, the effects of DDR1a in proliferation and response to cytotoxicity induced by 5-FU were further evaluated.

It was reported that cell proliferation affects drug resistance. Reducing cell apoptosis or speeding up cell proliferation activity could enhance drug resistance [26]. In our study, the data showed that 5-FU suppressed the proliferation of LoVo cells in a concentration- and time-dependent manner. Moreover, overexpressed DDR1a was found to be insensitive to 5-FU, whereas the knocked down DDR1a was found to be sensitive to 5-FU (IC_50_: 16.15 vs 2.59 µM at 48 h). Thus, the knockdown of DDR1a inhibited the cell proliferation and increased the sensitivity to 5-FU. Sensitivity of cells to chemotherapy might be estimated by examining apoptosis. Thus, Hoechst 33,342 staining and TUNEL assay were performed to evaluate apoptosis induced by 5-FU in transfected LoVo cells. After 5 μM 5-FU treatment for 48 h, the transfected cells displayed marked apoptotic characteristics, including cell shrinkage, cytoplasmic vacuolation, and nuclear fragmentation. Alternatively, these phenomena were more common in the DDR1a knockdown group. This conclusion is consistent with the observations drawn from the TUNEL assay. In addition, knockdown of DDR1a facilitated cytochrome C release from mitochondria and activated caspase-3. Therefore, we conclude that DDR1a most likely enhances LoVo sensitivity to 5-FU by inhibiting cell proliferation and inducing apoptosis. However, the specific mechanism still remains unclear.

Previous studies noted that the activation of PI3K/AKT/mTOR and Wnt/*β*-catenin [27,28] signaling pathways protects CRC and other tumors from toxic effects caused by chemotherapy agents [29,30]. Our results showed that DDR1a overexpression upregulated the expressions of PI3K and MDM2, while DDR1a knockdown elevated the BAX/Bcl-2 ratio. 5-FU treatment decreased the expression of PI3K as well as the p-AKT/AKT ratio in both DDR1a^high^ and shDDR1a cells. However, BAX/Bcl-2 levels in the shDDR1a group were still higher than those in the control. Therefore, knockdown of DDR1a promoted apoptosis induced by 5-FU in LoVo cells via PI3K/AKT/Bcl-2 pathway. The current study concurs with previous research [4,31,32].

## Conclusions

To summarize, DDR1a may be a critical factor in enhancing the sensitivity of LoVo cells to 5-FU and promoting apoptosis after 5-FU treatment by activating PI3K/AKT/Bcl-2 signaling pathway. The present study had some limitations; the LoVo cell lines may be more suitable for studying drug resistance due to its genetic background. However, a single-cell line could not fully uncover the function and associated mechanisms of DDR1a in 5-FU chemoresistance. Therefore, further research is necessary.

## Data Availability

The datasets used and/or analyzed during the current study are available from the corresponding author on reasonable request.
